# Predictive factors of radioactive iodine therapy failure in hyperthyroidism: a retrospective study of 171 patients

**DOI:** 10.11604/pamj.2025.52.87.49292

**Published:** 2025-10-30

**Authors:** Skander Chaabouni, Imen Meddeb, Marwa Somai, Asma Krir, Mehdi Mrad, Afef Bahlous, Aida Mhiri

**Affiliations:** 1 *Institut Pasteur de Tunis*, Clinical Biochemistry and Hormonology Laboratory, *Gouvernorat de Tunis*, Tunis, Tunisia; 2 Salah-Azaïz Institute, Nuclear Medicine Department, *Gouvernorat de Tunis*, Tunis, Tunisia

**Keywords:** Hyperthyroidism, radioactive iodine, treatment failure, predictive factors, biomarkers

## Abstract

**Introduction:**

radioiodine therapy with iodine-131 is a standard treatment for hyperthyroidism. Nevertheless, the variability in individual response underscores the need to identify reliable predictors of treatment failure. The study aimed to determine the clinical and biological factors associated with the failure of the first course of radioactive iodine therapy in hyperthyroid patients.

**Methods:**

this was a retrospective cohort study conducted between 2012 and 2018, including 171 patients treated for hyperthyroidism with radioiodine therapy at the Salah Azaiez Institute. Demographic, clinical, biological, and therapeutic data were collected and analysed.

**Results:**

the median age was 49 years, with a female predominance (sex ratio: 0.41). Graves' disease accounted for 73.7% of cases, and resistance to antithyroid drugs was the main indication for radioiodine therapy (68%). A fixed dose of iodine-131 was administered (mean: 14 ± 2.6 mCi). Treatment failure after the first course occurred in 22.2% of patients (n=38). Failure was significantly associated with higher administered doses (p=0.007), TSH receptor antibody positivity (p=0.005), and the presence of ophthalmopathy (p=0.013).

**Conclusion:**

failure after radioiodine therapy appears to be influenced by biological markers (TSH receptor antibodies) and specific clinical factors (ophthalmopathy). These results support individualised dosing strategies and highlight the need for multicenter studies to standardise treatment protocols in nuclear medicine centers in Tunisia.

## Introduction

Hyperthyroidism (HT) is a common endocrinopathy, defined by elevated free thyroid hormones and resulting in a thyrotoxic syndrome whose clinical manifestations vary according to the underlying aetiology [1,2]. It can occur at any age and may lead to significant systemic and psychosocial consequences, warranting prompt and appropriate management. Diagnosis is based on hormonal assays, complemented by the detection of autoantibodies to determine the aetiology, most commonly Graves' disease. Treatment options include antithyroid drugs, surgery, and radioactive iodine (^131^I), the latter increasingly used as a first-line therapy in certain contexts [3]. However, the response to radioiodine therapy varies considerably among patients, emphasising the need to identify reliable clinical and biological predictive factors [4,5]. In the absence of large-scale local data, we conducted this study in the Department of Nuclear Medicine at the Salah Azaiez Institute, aiming to determine the clinico-biological factors associated with the response to ^131^I therapy in patients with hyperthyroidism.

## Methods

**Study design:** this was a retrospective, observational, single-centre study conducted between January 2012 and December 2018.

**Setting:** the study was conducted at the Department of Nuclear Medicine, Salah Azaiez Institute, Tunis, Tunisia, between January 2012 and December 2018. This semi-public tertiary care centre receives patients from across the country. All patients referred for radioactive iodine therapy during this period were included in the study. Clinical, biological, and imaging data were retrospectively collected from medical records using a standardised collection form. Follow-up assessments were performed at 3 and 6 months after the first course of radioactive iodine therapy, including measurement of thyroid-stimulating hormone (TSH) and free thyroxine (FT4) levels, to evaluate treatment response.

**Participants:** all patients treated for hyperthyroidism with radioactive iodine (iodine-131) who had documented clinical and biological follow-up after therapy were included. Hyperthyroidism was confirmed by suppressed TSH with elevated T3 and/or free T4 levels. Exclusion criteria were: concomitant thyroid malignancy, absence of significant tracer uptake on thyroid scintigraphy, lack of pre- or post-therapeutic biological follow-up, incomplete medical records, and age under 18 years. A total of 171 patients were included.

**Variables:** the primary outcome was resistance to radioiodine therapy, defined as persistent hyperthyroidism (TSH below reference values) at 6 months after the first course. Secondary outcomes included early response (TSH above reference at 3 months) and favourable response (TSH above reference at 6 months). Explanatory variables: age, sex, medical history, duration of symptoms, aetiology of hyperthyroidism (Graves', nodular, iatrogenic, other), prior antithyroid drug therapy (type, dose), clinical manifestations (weight loss, heat intolerance, cardiovascular, neuropsychiatric, gastrointestinal symptoms), presence of exophthalmos, administered radioiodine dose, and scintigraphic findings.

**Data sources/measurement:** data were extracted from medical records using a standardised collection form. Thyroid scintigraphy was systematically performed on the day of iodine administration, approximately 20 minutes after intravenous technetium-99m injection, to confirm tracer uptake and guide therapeutic decisions. Patients fasted for at least two hours before therapy to optimise iodine bioavailability. Radioiodine was administered orally, either as a capsule or liquid solution, with sufficient water. The administered activity was determined based on hyperthyroidism severity and scintigraphic pattern, following the French Society of Nuclear Medicine recommendations.

**Bias:** selection bias was minimised by including all eligible patients during the study period. Information bias was reduced by using standardised methods for clinical, biological, and imaging assessments.

**Study size:** no a priori sample size calculation was performed; all eligible patients were included in the study.

**Quantitative variables:** continuous variables such as age were expressed as mean ± standard deviation or median [interquartile range], depending on distribution. Follow-up time points (3 and 6 months) were analysed for early and favourable response evaluation.

**Statistical methods:** data were entered into Excel and analysed using SPSS version 20.0. Comparisons were made using Student's t-test or Mann-Whitney test for continuous variables, and chi-square or Fisher's exact test for categorical variables, as appropriate. Multivariate logistic regression was used to identify factors independently associated with treatment failure (p < 0.05 considered significant). Correlations between continuous variables were assessed using Pearson or Spearman tests. Data confidentiality was maintained throughout the study.

## Results

**Participants:** a total of 171 patients treated with radioactive iodine for hyperthyroidism were included. Patients were excluded if they had concomitant thyroid malignancy, absent scintigraphic uptake, incomplete follow-up, or age under 18 years. The flow of participants through the study is summarised in Table 1.

**Table 1:** measurement techniques and reference values of studied biological parameters

Characteristic	Value / number	Percentage (%)
Number of patients included	171	100
Mean age (± SD)	48.8 ± 16.4 years	-
Sex ratio (M/F)	0.41	-
**Clinical symptoms**		
Weight loss + fatigue	107	62
Heat intolerance (clinically confirmed)	111	64.9
Exophthalmos	29	17.5
Respiratory signs	31	18.1
Neuropsychological disorders	116	67
Digestive disorders	15	8
**Hormonal profile before treatment**		
Median FT4 [IQR]	26 pmol/L [16-50]	-
Elevated FT4	108	63.2
Median TSH [IQR]	0.03 μIU/mL [0.03-0.05]	-
Suppressed TSH (<0.1 μIU/mL)	166	97
**Immunological status**		
Positive ATPO	89	52
Positive anti-TSHR	71	41.5
**Previous ATD treatment**	133	77
**Mean first-dose of I-131**	14 ± 2.6 mCi	-
**Failure of first cure**	38	22.2

Abbreviations: FT4 = free thyroxine; TSH = thyroid-stimulating hormone; TRAb = TSH receptor antibodies; ATPO = anti-thyroperoxidase antibodies; ATD = antithyroid drugs; mCi = millicurie

**Descriptive data:** the mean age of the cohort was 48.8 ± 16.4 years, with a male-to-female sex ratio of 0.41 (mean age: 47.6 ± 16.8 years for women, 51.5 ± 15.1 years for men). Exophthalmos was observed in 17.5% of patients (n=29), one-third of whom required corticosteroid therapy. Baseline hormonal assessment showed thyrotoxicosis in the majority of cases: median free T4 (FT4) was 26 pmol/L [16-50], with 63.2% (n=108) exhibiting elevated FT4. Median TSH was 0.03 µIU/mL [0.03-0.05], with 97% (n=166) of patients having TSH < 0.1 µIU/mL. Thyroid autoantibodies were positive in 52% for anti-thyroperoxidase (anti-TPO, n=89) and 41.5% for TSH receptor antibodies (TRAb, n=71). The main indication for radioiodine therapy was antithyroid drug resistance (68%), followed by recurrence (6%), drug-induced toxicity (4%), and clinician preference for radical treatment (22%). Before therapy, 77% (n=133) had received antithyroid drugs.

**Outcome data:** the mean administered dose of iodine-131 for the first course was 14 ± 2.6 mCi (range 8-25 mCi). A second course was required in 21% (n=36) of patients, with a mean dose of 14.4 ± 3.1 mCi. Aetiology-specific doses were as follows:

***Graves' disease (n=126):*** mean first-course dose 13.4 ± 2.1 mCi; 22.2% required a second course.

***Toxic nodule (n=3):*** mean dose 15.6 ± 3.3 mCi; no second course required.

***Multinodular goiters (n=17):*** initial mean dose 15.7 ± 3.5 mCi; 23.5% required a second course (18.25 ± 4.7 mCi).

***Basdowe goiters (n=25):*** first-course mean dose 15.52 ± 2.43 mCi; 16% required a second course (17.5 ± 2.8 mCi).

**Main results:** biological follow-up at 3 months post-first course (n=97, 57%) showed early response (hypothyroidism) in 38% (n=37), normal TSH in 17%, and persistent hyperthyroidism in 45%. At 6 months (n=124), 70% of patients achieved a favourable response, while 30% experienced treatment failure (persistent hyperthyroidism). One patient lost to follow-up without TSH measurement at 6 months was considered a good responder. Predictive factors of treatment failure at 6 months after the first course are summarised in Table 2.

**Table 2:** summary of factors associated with response to radioiodine therapy

Factor	Categories	Favorable Response (n=133)	Resistance (n=38)	p-value	Interpretation
**Age (years)**	<35 (n=42)	25 (59.5%)	17 (40.5%)	NS	No significant correlation (p=0.13)
	35–49 (n=47)	40 (85.1%)	7 (14.9%)		
	50–59 (n=40)	34 (85%)	6 (15%)		
	>60 (n=42)	34 (80.9%)	8 (19.1%)		
**Gender**	Female	97 (80%)	24 (20%)	NS	No significant difference
	Male	38 (76%)	12 (24%)		
**Ophthalmopathy**	Present	12 (41%)	17 (59%)	0.013	Significant association, OR = 3.7 (95% CI: 1.31-10.4)
**Respiratory signs**	Present	26 (84%)	5 (16%)	NS	Not significant
**Cardiovascular signs**	Present	85 (76.5%)	26 (23.5%)	NS	Not significant
**Digestive signs**	Present	9 (60%)	6 (40%)	0.094	Not significant
**ATD therapy**	Yes	99 (74.5%)	34 (25.5%)	NS	No significant influence
**Etiology**	Graves’ disease (n=126)	96 (76%)	30 (24%)	NS	No significant difference
	Toxic nodule (n=3)	3 (100%)	0		
	Multinodular goiter (n=17)	13 (76%)	4 (24%)		
	Basdowe goiter (n=25)	21 (84%)	4 (16%)		
**Hormonal status**	FT4 (pmol/L)	21.47 [12.18; 45.6]	29 [20; 48]	NS	No correlation
	TSH (μIU/mL)	0.03 [0.03; 0.05]**	0.03 [0.01; 0.03]***	0.007*	Significant correlation (univariate)
**Immunological status**	TRAb +	46 (65%)	25 (35%)	0.005	Significant association, OR = 3.79 (95% CI: 1.5-9.5)
	ATPO +	65 (73%)	24 (27%)	NS	No significant association
**First-course RAI dose (mCi)**	Median [P25; P75]	12 [12; 15]	15 [12; 15]	0.007	Significant negative correlation, OR = 0.778 (95% CI: 0.649-0.933)

Abbreviations: FT4 = free thyroxine; TSH = thyroid-stimulating hormone; TRAb = TSH receptor antibodies; ATPO = anti-thyroperoxidase antibodies; ATD = antithyroid drugs; mCi = millicurie. *Significant in univariate analysis. **Median equal to 25th percentile (values at detection limit). ***Median equal to 75th percentile (same reason).**Other analyses:** radioiodine therapy resistance was observed in 22.2% of patients (n=38). Multivariate analysis of factors influencing treatment response showed that, clinically, only the presence of ocular manifestations (exophthalmos) was significantly associated with resistance to therapy (p=0.013). Biologically, TSH receptor antibody (TRAb) positivity was correlated with therapy resistance (p=0.005). In univariate analysis, baseline TSH was significantly lower in patients who failed therapy (p=0.007), but this association did not persist in multivariate analysis. Therapeutically, the administered activity of iodine-131 during the first course was significantly associated with resistance (p=0.007). Other sociodemographic, clinical, and biological factors were not significantly associated with treatment response.

## Discussion

**Table 3** summarises the most relevant studies evaluating predictive factors for response to radioactive iodine therapy in hyperthyroidism. This represents the largest national study evaluating the response to fixed-dose iodine-131 therapy in hyperthyroidism.

**Table 3:** major studies evaluating predictive factors for response to radioactive iodine therapy in hyperthyroidism

Author (Year)	Number of Patients	Study Type	Significant Factors Identified
Allahabadia *et al*. (2001)	926	Retrospective	ATD pretreatment, FT4 levels, iodine dose
Schneider DF *et al*. (2014)	325	Retrospective	TRAb positivity = risk of failure
El Issami *et al*. (2011)	280	Retrospective	Iodine dose
Sellem A *et al*. (2020)	54	Descriptive	Iodine dose, exophthalmia, ATD pretreatment
Boelaert K *et al*. (2008)	1278	Retrospective	FT4 levels, iodine dose, ATD pretreatment, orbitopathy
Yassine I *et al*. (2015)	35	Retrospective	TRAb levels, exophthalmia

**Summary of main findings:** our study included 171 patients with hyperthyroidism referred for radioactive iodine therapy at the Salah Azaiez Institute. The main indications were resistance to antithyroid drugs (68%, n=116), recurrence of hyperthyroidism (6%, n=10), and drug-induced toxicity (4%, n=7). The remaining patients (22%, n=38) were referred according to the treating physician's choice for definitive isotopic treatment. Graves' disease was the most frequent aetiology, affecting 73.7% of the study population, with a clear female predominance across all etiologies. Fixed doses of radioactive iodine were administered, with a mean of 14 ± 2.6 mCi.

Treatment failure after the first course of radioactive iodine was observed in 22.2% of patients (n=38). Multivariate analysis showed that, clinically, only the presence of ocular manifestations was significantly associated with therapy resistance (p=0.013). Biologically, TSH receptor antibody (TRAb) positivity was correlated with resistance (p=0.005). In univariate analysis, baseline TSH was significantly lower in patients who failed therapy (p=0.007), but this association did not persist in multivariate analysis. Therapeutically, the administered activity of iodine-131 during the first course was significantly associated with resistance (p=0.007). Other sociodemographic, clinical, and biological factors were not significantly associated with treatment response.

**Comparison with the literature:** the mean age in our cohort was 48.8 ± 16.4 years, comparable to reports by Schneider *et al*. (43.4 ± 15.5 years) and Issami *et al*. (49.5 years) [6,7]. The aetiology of hyperthyroidism may vary with age: Graves' disease commonly occurs between 30 and 50 years, whereas toxic nodular goiter is more frequent after 50 years, and toxic adenoma tends to occur at younger ages [8,9]. Our series, dominated by Graves' disease, primarily involved patients aged 30-60 years.

No significant correlation was observed between age and response to iodine-131 (p=0.13), which may be explained by the use of fixed doses according to aetiology, itself influenced by age. Resistance was more frequent in patients under 35 years (40.5%), followed by those over 60 years (19.1%), whereas patients aged 35-49 years showed the highest response rate (85.1%). Several studies suggest that advanced age is associated with higher success rates [10-12].

As expected, hyperthyroidism was more frequent in women (F/M sex ratio = 2.43), consistent with previous reports [13-15], with no significant difference in treatment response between sexes. The most common clinical signs were general, neurological, and cardiovascular, in agreement with other series [6,15]. Orbitopathy was significantly associated with resistance to radioiodine therapy (p=0.013), as previously reported [16]. It may interfere with radioiodine uptake due to inflammation and anatomical changes [16]. Tachycardia has also been associated with treatment failure (p<0.01) [6].

Regarding aetiology, no significant correlation with treatment response was observed, consistent with other studies [6,13], although some authors suggest relative radioresistance in nodular forms [17-20], possibly due to persistent autonomous tissue with reduced uptake [17]. The predictive role of FT4 remains debated. Some studies report that elevated FT4 is associated with an increased risk of failure [3,9,18], but our findings, as well as those of others [21,22], did not show a significant relationship. Conversely, low TSH was associated with a higher rate of failure (significant univariate correlation), supporting data from Schneider [6]. Elevated TSH would enhance iodine uptake, thereby improving treatment efficacy. However, this correlation was not observed in studies by Aizawa *et al*. and Husseni *et al*. [23,24].

In our cohort, TRAb positivity in patients with Graves' disease was 48%, similar to several other studies [25], although Yassine *et al*. reported 100% positivity [19]. As classically reported, all patients with toxic nodules were TRAb-negative [25], while results for other etiologies remain variable [26]. Studies by Mathew John highlighted the diagnostic value of TRAb in hyperthyroidism [27]. In our study, TRAb positivity was significantly associated with resistance to radioiodine therapy (p=0.005), with an odds ratio of 3.79 (95% CI: 1.5-9.5), consistent with multiple publications [28,29]. This resistance may be explained by chronic stimulation of TSH receptors leading to glandular remodelling, such as fibrosis or inflammation, limiting iodine uptake [30]. However, other studies suggest an inverse association, linking TRAb positivity to better treatment outcomes [23]. Regarding anti-thyroperoxidase antibodies (anti-TPO), our study did not find a significant association with response to iodine-131, in agreement with previous reports [30]. Although frequently present in thyroid disorders, anti-TPO antibodies do not appear to directly influence treatment sensitivity.

Our cohort study revealed that all patients exhibited an unfavourable course under antithyroid drugs (ATD), defined by persistent or recurrent hyperthyroidism, or the occurrence of adverse effects. However, no significant correlation was observed between ATD use and response to radioiodine therapy, consistent with previous studies [31,32]. Some authors, nevertheless, have highlighted a potential radioprotective effect of ATDs, increasing the risk of therapeutic failure, particularly when administered immediately before the treatment [25]. This interference may result from a reduced radioactive iodine half-life, leading to suboptimal tissue exposure [33]. The type of ATD could also play a role, with some studies implicating specifically imidazoles [3], and others thio-uracil derivatives [34]. Furthermore, prolonged ATD use appears to be a factor of resistance in several studies [21]. Walter *et al*. recommend discontinuation at least one week before the treatment to improve its efficacy [35], a practice that was systematically applied in our series.

In this study, the main indication for radioiodine therapy was resistance to ATDs, as reported in several other series [2,4]. In other studies, intolerance or adverse effects of ATDs were the most frequent reasons for resorting to this therapy [4,7]. According to the National Institute for Health and Care Excellence (NICE) guidelines, radioiodine therapy is indicated as first-line treatment in thyrotoxicosis when ATDs are contraindicated or ineffective [36]. Since its introduction in 1941 [37], various therapeutic protocols have been proposed. In practice, the therapeutic target differs among centres, and no clear consensus exists regarding the optimal dose. Two strategies coexist: fixed doses based on aetiology, and calculated doses according to gland size and iodine uptake. Comparative studies of these approaches have produced conflicting results [38].

In our study, the nuclear medicine team at ISA used a standardised regimen, mainly based on aetiology. During the early years, adjustments were occasionally made according to age, hyperthyroidism severity, and socioeconomic context. In line with the latest American Thyroid Association (ATA) guidelines, a fixed and sufficient dose of iodine-131 (typically 10-15 mCi for autoimmune hyperthyroidism) should be administered in a single session [38]. These recommendations were followed in our series, with a mean dose of 14 mCi, comparable to that used in other studies [2,3]. Notably, we observed a negative correlation between the administered dose and therapeutic success (p<0.007; OR=0.778), in contrast with most previous studies reporting better response rates with higher doses, particularly in Graves' disease [2,25], as well as in toxic nodules and multinodular goiters [3,39,40]. This discrepancy may be explained by relative underdosing observed in our population for nodular etiologies, due to logistical constraints limiting access to sufficiently high activities. Treatment efficacy is generally proportional to the delivered absorbed dose, with recommended levels varying by aetiology: 130-200 Gy for uninodular goiters and 80-130 Gy for multinodular goiters [39]. Despite higher doses administered to patients with nodular etiologies, these remained below optimal thresholds. This situation could explain the more frequent failures observed in these subgroups compared to autoimmune forms.

The relationship between administered iodine dose, aetiology, and radioiodine therapy efficacy remains controversial. This variability likely results from differences in epidemiological profiles and etiological distribution among studies. In particular, the question of whether toxic nodules and multinodular goiters have higher failure rates than Graves' disease, and thus require higher doses, remains open [40,41]. Other factors should also be considered, such as renal clearance, which is reduced in renal insufficiency, or prior ATD treatment, both of which can alter the pharmacokinetics of radioactive iodine and thereby affect therapeutic efficacy.

**Limitations:** this study has several limitations. It is a single-centre, retrospective study, which may limit the generalizability of the findings. Some clinical and biological data were incomplete, and a few patients were lost to follow-up. Additionally, follow-up was limited to six months after the first course, preventing assessment of late hypothyroidism or delayed therapy failures. Variability in laboratory assay techniques across centres may also affect the comparability of results. Despite these limitations, all eligible patients during the study period were included, and standardised treatment and follow-up protocols were applied.

**Clinical implications and perspectives:** our study, conducted on a large national population-the largest to date in Tunisia-provides new insights into the relationship between administered iodine-131 activity and treatment efficacy in hyperthyroidism. The results emphasise the importance of adjusting administered doses to minimise the risk of treatment failure and optimise patient management. These findings support the need for prospective multicenter studies with standardised protocols, enabling better individualisation of therapy according to local practice and patient characteristics.

## Conclusion

Radioiodine therapy represents an effective, simple, and minimally invasive treatment option for hyperthyroidism. However, treatment response remains heterogeneous. In our study, several factors were identified as being associated with an increased risk of therapeutic failure, including orbitopathy, an initially suppressed TSH, and positivity for anti-TSH receptor antibodies. These findings support the implementation of an individualized strategy that takes into account each patient's clinico-biological profile to optimize the likelihood of treatment success.

### What is known about this topic

- Radioiodine therapy with iodine-131 is a widely used and effective treatment for hyperthyroidism, but treatment response remains heterogeneous;
- Several clinical and biological factors, such as disease type, thyroid size, and antibody status, have been suggested as potential predictors of treatment outcome, though findings remain inconsistent across studies.

### What this study adds

- This study identifies higher administered doses of iodine-131 (p=0.007), TSH receptor antibody positivity (p=0.005), and the presence of ophthalmopathy (p=0.013) as significant predictors of radioiodine therapy failure after the first course;
- It emphasises the need for individualised dosing strategies rather than a fixed-dose approach, and calls for multicenter studies to harmonise treatment protocols in Tunisia.
